# Characterization of coumarin-6 polycrystalline films growth from vacuum deposition at various substrate temperatures

**DOI:** 10.1038/s41598-018-34813-w

**Published:** 2018-11-13

**Authors:** Ko-Lun Chen, Hui-Ting Liu, Jang-Hung Yu, Yung-Hsiang Tung, Yun-Syuan Chou, Chun-Chuen Yang, Jyh-Shyang Wang, Ji-Lin Shen, Kuan-Cheng Chiu

**Affiliations:** Department of Physics and Center for Nanotechnology, Chung Yuan Christian University, Chungli District, Taoyuan City, 32023 Taiwan

## Abstract

Coumarin-6 polycrystalline films were fabricated from vacuum deposition at various substrate temperatures *T*_*sub*_ from 106 to 178 °C with a fixed source temperature of 185 °C. Because of its slenderer and more asymmetric structure, the adhered coumarin-6 molecule on top of the growing interface encounters a larger steric energetic barrier of 0.92 eV as estimated from the Arrhenius plot of growth rate versus 1/*T*_*sub*_. From top-view SEM pictures, the as-deposited coumarin-6 thin films exhibit a twisted pattern and a kinematic roughness for *T*_*sub*_ < 150 °C; while clear facets emerge for *T*_*sub*_ ≥ 150 °C due to the increase of surface diffusion energy of the adhered molecules. From XRD analysis, besides the confirmation of the triclinic structure two anomalous peaks observed at 2*θ* ~ 9.007° and 7.260° are explained due to the co-existence of N- and S-coumarin-6-isomers within the crystalline grains. Furthermore, for coumarin-6 polycrystalline films deposited at *T*_*sub*_ = 150 °C with high crystallinity of the constituent grains, the bandgap determined from optical transmission is around 2.392 eV; and from photoluminescence spectra, the fitted four emission components are assigned to the Frenkel and charge transfer excitons recombination with participation of molecular vibrational states.

## Introduction

The small organic molecule of 3-(2-benzothiazolyl)-7-(diethylamino)coumarin (C_20_H_18_N_2_O_2_S, named as coumarin-6 or coumarin-540)^1^ has been known and utilized as one of the fluorescent dyes for staining organelles or materials used in medicine^2,3^, and for high-gain medium in tunable and amplifier lasers^4–6^. Moreover, after being successfully introduced as an efficient dopant in organic light emitting diodes^7^, coumarin-6 has also attracted much interest in optoelectronic applications^8–13^. Coumarin-6 is a molecular chromophore with high quantum efficiency. While dissolved in various solvents, the absorption maximum ranged from 2.713 eV (in methanol or ethanol) to 2.912 eV (in n-hexane) and photoluminescence (PL) with peak emission maximum from 2.460 eV (in methanol) to 2.702 eV (in n-hexane) were reported^5^. As crystallized from supersaturated solutions, coumarin-6 tiny single crystal has a triclinic structure with lattice parameters *a* = 8.962 Å, *b* = 11.136 Å, *c* = 8.922 Å, *α* = 95.14°, *β* = 104.50°, and *γ* = 86.74° ^1,14^. Coumarin-6 molecular crystal possesses a relatively high melting point around 220 °C, a high stability of surface morphology, and an efficient light emission, and hence can be considered as an important organic material for optoelectronic devices. However, for acting as a dye or dopant in optoelectronic devices, only the molecular characters of coumarin-6 are focused. While in organic multilayer optoelectronic devices, a systematic study on the fabrication and characterization of coumarin-6 polycrystalline or amorphous thin films is prerequisite.

Vacuum deposition (*i.e*., deposition by thermal sublimation in vacuum) is one of the most common techniques adopted for depositing small organic molecular thin films if these molecules can be sublimed at an appropriate source temperature (*T*_*sou*_) without decomposition^7,15–17^. To obtain a significant growth rate, the vapor pressure from source powder governed by *T*_*sou*_ has to be high enough. The *T*_*sou*_-dependence of vapor pressure can be determined from the rate of mass loss per unit area by using a conventional thermogravimetric analysis (TGA)^18,19^. Besides, the substrate temperature (*T*_*sub*_) governs the surface kinetics of the adhered molecules, including adhesion, surface diffusion, and re-evaporation of the incoming molecules onto the substrate surface as well as the growing interface. The difference between *T*_*sou*_ and *T*_*sub*_ corresponds to the degree of supersaturation which affects the nucleation and subsequent growth^20–26^. The proper values of *T*_*sub*_ corresponding to a fixed *T*_*sou*_ can be experimentally determined from the concept of constitutional supersaturation (CSS), and the effects of *T*_*sub*_ on the size, orientation, and crystallinity of the constituent grains and on the growth rate of various small organic molecular polycrystalline films were reported^21–25^.

**Figure 1:**
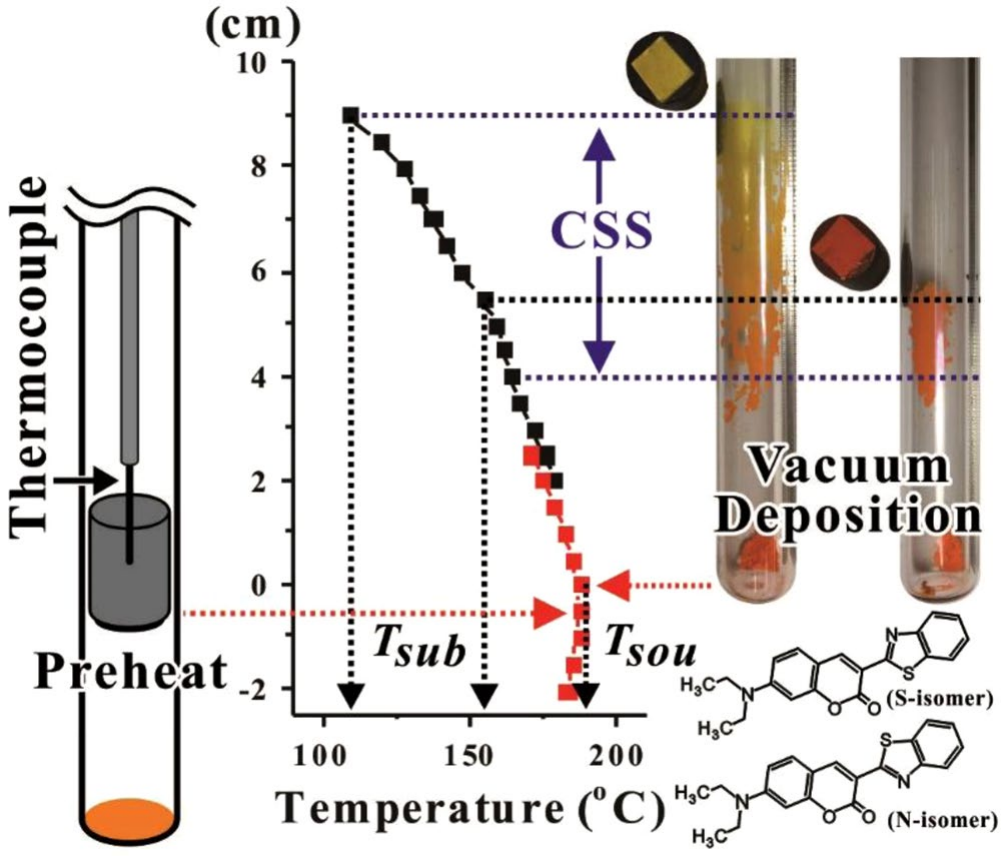
Growth ampoule under pre-heat process and after vacuum deposition with CSS region are schematically demonstrated. Two coumarin-6 films (on ITO substrate with graphite susceptor) deposited with different *T*_*sub*_ are illustrated. The S- and N-isomers by turning the benzothiazolyl moiety upto 180° are depicted.

Within a coumarin-6 molecular crystal, as demonstrated in Fig. 1, the coumarin moiety is nearly coplanar with the benzothiazolyl moiety (*i.e*., the pyrone ring in the essentially planar structure of coumarin moiety makes a small angle of 2.30° with the thiazolyl ring and 3.29° with the benzene ring in the benzothiazolyl moiety, respectively)^1^. However, in coumarin-6 crystalline grains, two conformational isomers (namely, S-isomer and N-isomer) should be distinguished by turning the benzothiazolyl moiety upto 180° such that the relative position of S or N atom in the benzothiazolyl moiety with respect to the O atom in the coumarin moiety is different. So far, there is no report in literature on these two isomers. Thus, the effects from these isomers on the properties of the as-grown coumarin-6 molecular solid films remain to be clarified.

In this work, coumarin-6 polycrystalline films on indium-tin-oxide (ITO) substrate were grown from vacuum deposition at various *T*_*sub*_ with a fixed *T*_*sou*_. The growth behavior of coumarin-6 polycrystalline films is studied in terms of an Arrhenius plot of growth rate versus 1/*T*_*sub*_. The corresponding steric energetic barrier *E*_*A*_ on the formation of coumarin-6 molecular solid films is discussed and compared with other small organic molecules with different space symmetries. In addition, the morphological, structural, and optical properties of the as-deposited coumarin-6 polycrystalline films with respect to *T*_*sub*_ are also systematically investigated with respect to the S- and N-isomers. This work, though of technological importance to the fabrication of coumarin-6 thin films for applications in organic multilayer devices, is also expected to clarify some fundamental problems of crystallization mechanisms, weak intermolecular interactions, and surface kinematic effects for the production of organic molecular solids.

## Results and Discussion

### Determining the deposition conditions from TGA and CSS

**Figure 2:**
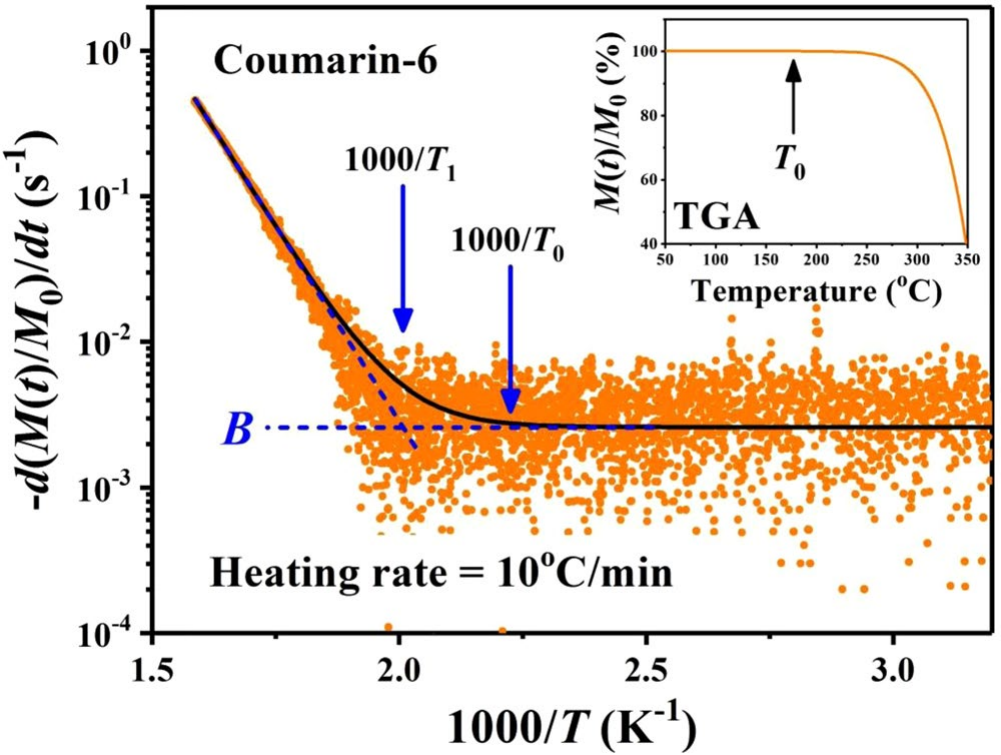
The rate of mass loss percentage, −*d*(*M*(*t*)/*M*_0_)/*dt*, versus 1000/*T* for coumarin-6 source powder. *T*_0_ (≅175 °C) indicates a threshold of sublimation loss, and *T*_1_ (≅224 °C) is associated with the melting point for coumarin-6 powder. The inset demonstrates the TGA curve with a heating rate of *dT*/*dt* = 10 °C/min.

The temperature (*T*)-dependence of equilibrium vapor pressure from the source powder plays an important role during the vacuum deposition process^20–25^. However, the data of vapor pressure over a wide enough *T* range for some small organic molecules are often difficult to obtain, and based on our literature survey the relevant information for coumarin-6 is not available. Thus, by using a conventional TGA, a method relating the vapor pressure with the rate of mass loss per unit area was adopted^18,19^. The inset in Fig. 2 demonstrates the TGA curve for coumarin-6 with a heating rate of *dT*/*dt* = 10 °C/min, and Fig. 2 represents the rate of mass loss percentage, −*d*(*M*(*t*)/*M*_0_)/*dt*, versus 1000/*T* (here *T* is in Kelvin scale), where *M*(*t*) is the mass at time *t* and *M*_0_ is the initial mass of the sample. The data in Fig. 2 could be fitted by a function of *A*×exp(−*H*_*F*_/*kT*) + *B*, where *H*_*F*_ is associated with the enthalpy of fusion, *k* is the Boltzmann constant, and *A* and *B* are constants^16–19^. For *T* < *T*_0_*, M*(*t*) remained nearly unchanged as depicted in the inset, thus the very low and rather noisy values of −*d*(*M*(*t*)/*M*_0_)/*dt* were represented by a background *B* value. At *T* = *T*_0_ (≅175 °C), the deviation of the fitted curve from the background *B* level indicated a threshold of sublimation loss. For *T* ≫ *T*_1_, the slope of the fitted straight line was associated with *H*_*F*_ (≅1.08 eV), and the intercept point of the straight line with the background *B* was related to the melting point (*T*_1_ ≅ 224 °C). To obtain a reasonable sublimation rate for thin film deposition, *T*_*sou*_ could be empirically selected around *T*_0_. In this work, *T*_*sou*_ = 185 ± 2 °C was chosen, then the side wall deposition corresponding to a CSS zone is demonstrated in Fig. 1. The CSS zone is a region where the actual vapor pressure exceeds the equilibrium vapor pressure such that the degree of supersaturation is large enough for nucleation and subsequent growth^21–25^. Based on the spreading of CSS zone, *T*_*sub*_ was varied from 106 to 178 °C, and the deposition period *τ* = 51 ± 1 min was chosen.

Usually, the growth modes in vacuum deposition can be categorized into mass-transport controlled regime and surface-kinetics controlled regime^20,26^. In this work with a high enough *T*_*sou*_ (>*T*_0_ ≅ 175 °C), the free space between source and substrate is nearly occupied with the convection of vaporized molecules with vapor pressure determined by *T*_*sou*_. Only near the substrate side a very thin diffusion boundary layer exists. As a CSS region is observed on sidewall near substrate side, the growth behavior on top of the substrate is dominated by surface-kinetics controlled regime. Consequently, the properties of the as-deposited molecular solid films are sensitively dependent on *T*_*sub*_.

### Surface morphology of coumarin-6 films fabricated at various *T*_*sub*_

**Figure 3:**
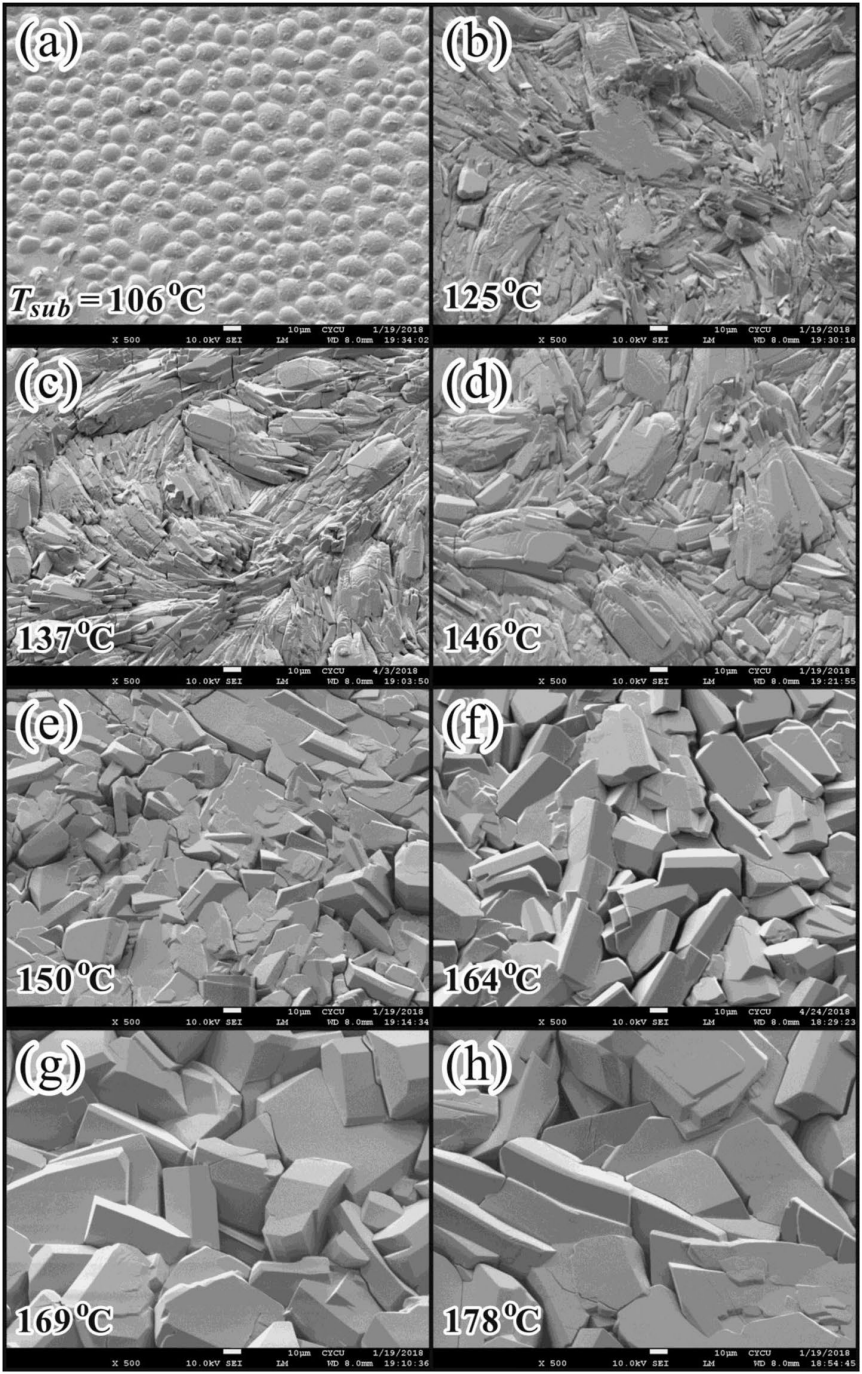
Top-view SEM images on the coumarin-6 polycrystalline films deposited at various *T*_*sub*_ with *T*_*sou*_ = 185 °C.

**Figure 4:**
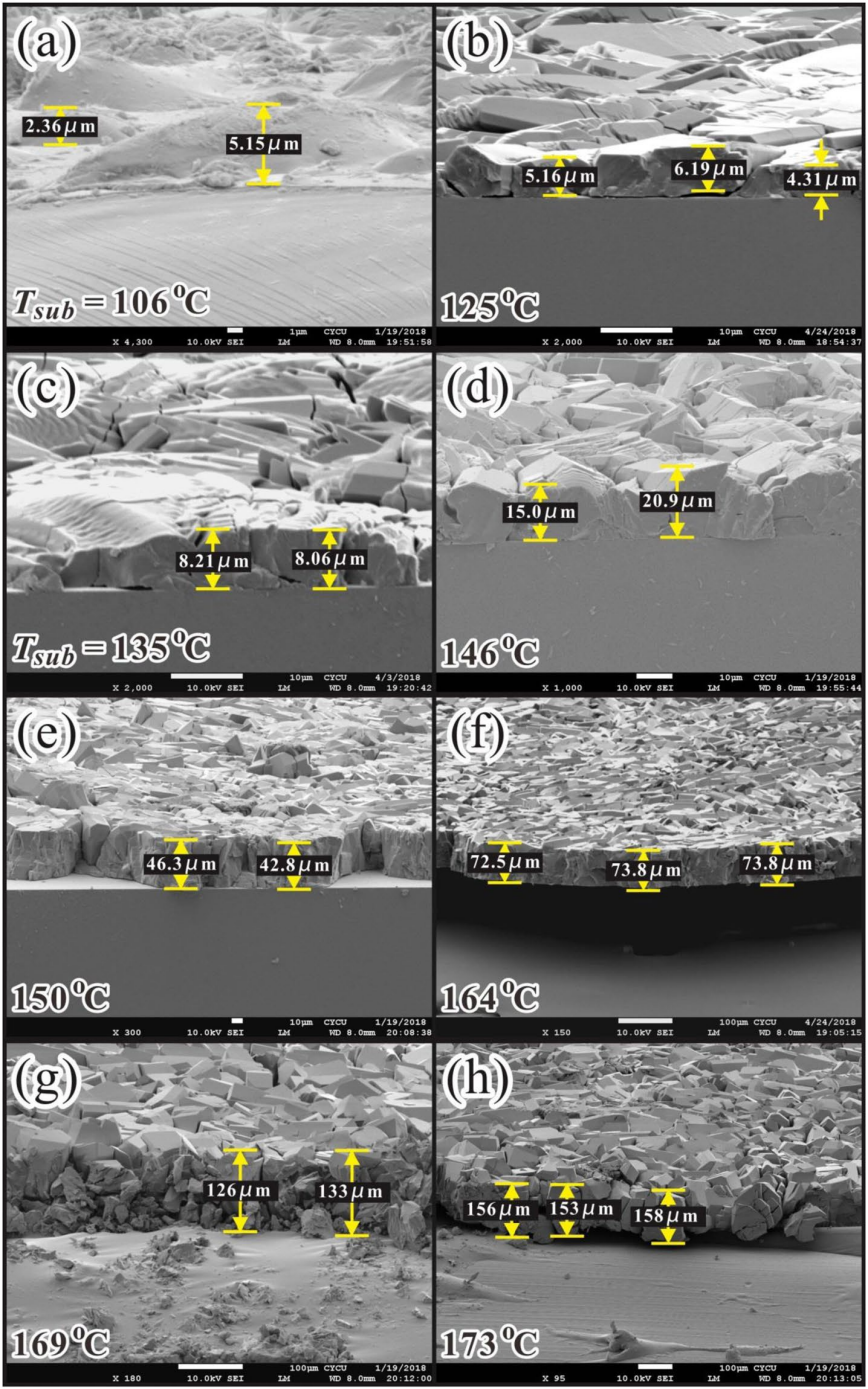
Side-view SEM images on the coumarin-6 polycrystalline films deposited at various *T*_*sub*_ with *T*_*sou*_ = 185 °C. The film thickness is revealed.

Figures 3 and 4 show the top- and side-view SEM pictures of coumarin-6 polycrystalline films deposited at various *T*_*sub*_, respectively. For a substrate located within the CSS region, the flux of incoming vaporized molecules is larger than the flux from re-evaporation, thus the net growth behavior is dominated by the surface-kinetics controlled regime^26^. Hence, both the adhesive probability of the incident molecules and the surface diffusion energy of the adhered molecules on the substrate as well as on the growing interface are increased with increasing *T*_*sub*_. From Figs. 3 and 4, for the lowest *T*_*sub*_ of 106 °C, the adhesive probability of the incoming coumarin-6 molecule on the substrate was not high enough, thus the coverage of the coumarin-6 thin film on the ITO substrate was incomplete. Until *T*_*sub*_ ≥ 125 °C, the coverage become complete, and from the side-view SEM pictures the average thin film thickness *L*_*D*_ was estimated. Furthermore, from the top-view SEM pictures as shown in Fig. 3, for 125 °C ≤ *T*_*sub*_ < 150 °C, the adhered coumarin-6 molecules on the interface do not have enough surface kinetic energy to re-arrange their relative conformation and to diffuse to the appropriate growth sites, thus the as-deposited coumarin-6 thin films exhibited a twisted pattern and a slightly kinematic roughness. Until *T*_*sub*_ ≥ 150 °C, clear facets corresponding to coumarin-6 crystalline grains emerged.

### Growth behavior of coumarin-6 thin films

**Figure 5:**
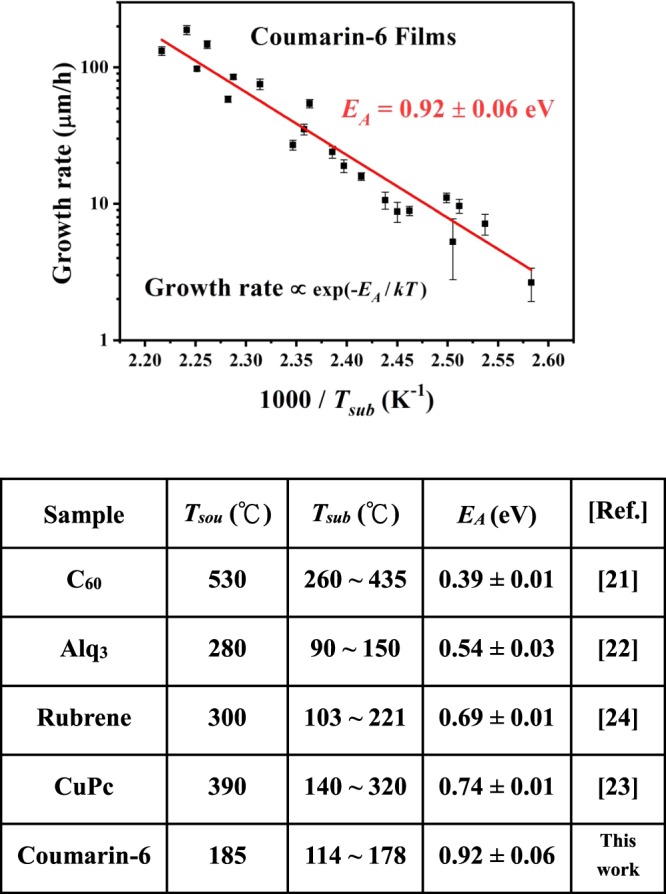
Arrhenius plot of growth rate versus 1000/*T*_*sub*_ for the coumarin-6 polycrystalline films deposited at various *T*_*sub*_ with *T*_*sou*_ = 185 °C. *E*_*A*_ obtained for some small organic molecular solid films vacuum deposited within the CSS region^21–24^ is summarized.

As shown in Fig. 4, the coverage of coumarin-6 solid films on ITO substrate become complete for *T*_*sub*_ ≥ 125 °C, and from the side-view SEM pictures the average thin film thickness *L*_*D*_ was estimated. Together with the deposition period *τ*, the growth rate of coumarin-6 polycrystalline films was obtained. The Arrhenius plot of growth rate versus 1000/*T*_*sub*_ (here *T*_*sub*_ is in Kelvin temperature scale) is demonstrated in Fig. 5. For an organic molecular crystal growth from vapor phase, the adhered molecules have to overcome a steric energetic barrier *E*_*A*_ in order to occupy the appropriate location (with more nearest neighbors) and the correct orientation (to maximize the intermolecular interactions)^26^. The corresponding *E*_*A*_ for coumarin-6 polycrystalline films evaluated from the Arrhenius plot was about 0.92 ± 0.06 eV. Together with our previous works for C_60_, Alq3, rubrene and CuPc^21–24^, the related parameters for these small organic molecular solid films deposited within the CSS region are summarized. Due to its slenderer and more asymmetric structure accompanied by a larger permanent dipole, the coumarin-6 molecules were expected to encounter a higher steric hindrance to occupy the appropriate location and correct orientation, and hence possessed a larger *E*_*A*_.

### XRD analysis of coumarin-6 films fabricated at various *T*_*sub*_

**Figure 6:**
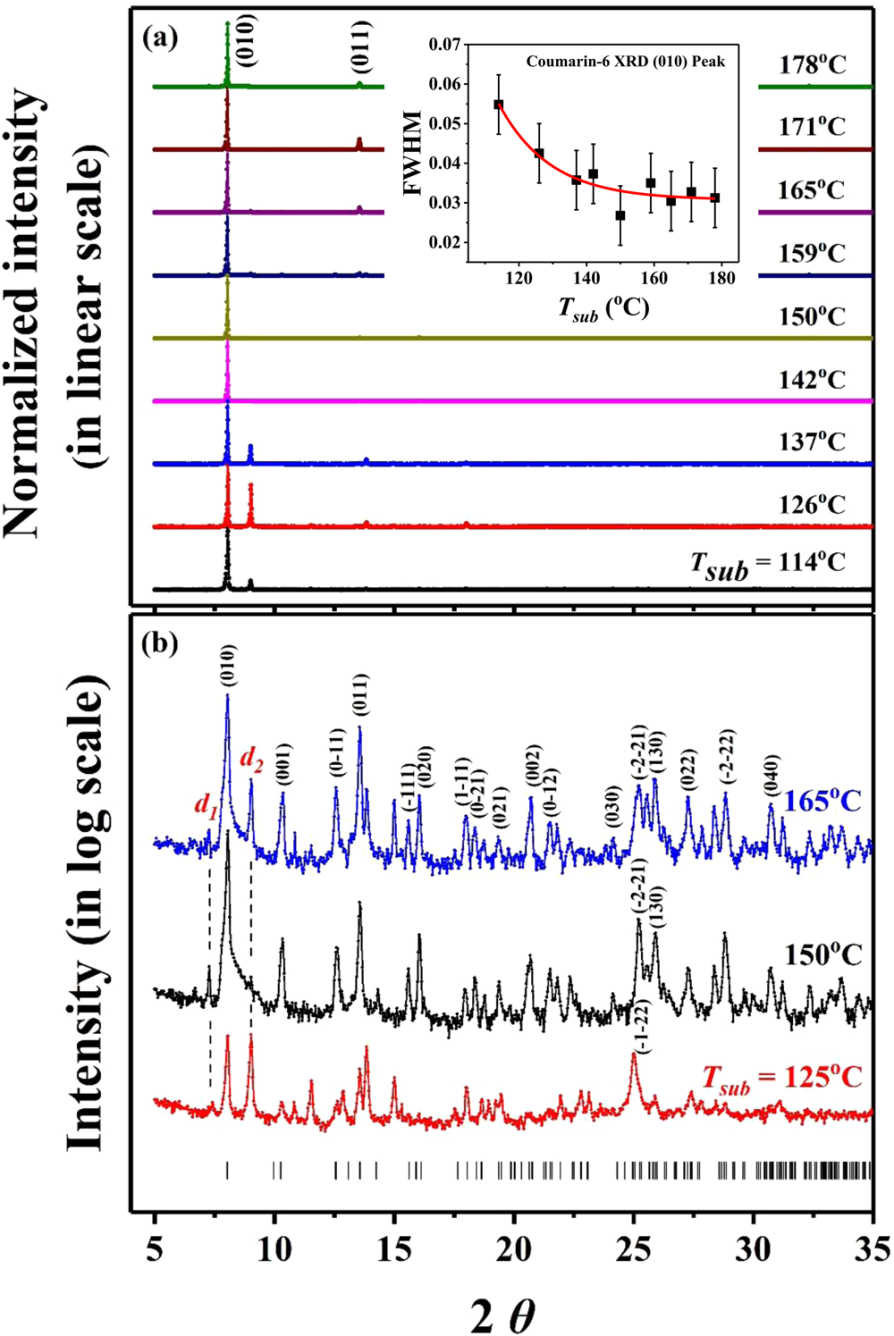
(**a**) Normalized XRD patterns (in linear scale) on the coumarin-6 polycrystalline films deposited at various *T*_*sub*_ with *T*_*sou*_ = 185 °C. Each curve is shifted up equal distance. The inset shows the variation of FWHM of the (010) peaks with respect to *T*_*sub*_. (**b**) XRD on the powder-samples taken from the films deposited at *T*_*sub*_ = 125, 150 and 165 °C. The XRD intensities are presented in logarithm scale, and the simulated peak positions from GSAS are shown at the bottom. The Miller indices of the main peaks are assigned based on COD ID 2003249^1^.

Figure 6 illustrates the XRD patterns for (a) the coumarin-6 polycrystalline films on top of ITO substrates in a normalized and linear scale and (b) the coumarin-6 powders taken from the as-deposited films in a logarithmic scale, separately. The Miller indices of the main peaks were assigned based on Crystallography Open Database for COD ID 2003249^1^. As depicted in Fig. 6(a), a protruding peak at 2*θ* = 8.039 ± 0.009° corresponding to the (010) peak was observed for all the coumarin-6 polycrystalline films fabricated at *T*_*sub*_ = 114~178 °C. This finding indicates that the preferred growth direction of these films on ITO substrate was parallel to the long axis of coumarin-6 molecular crystal. As shown in the inset of Fig. 6(a), the full width at half maximum (FWHM) of these (010) peaks were analyzed by a Gaussian fit with consideration of detector resolution under the same scanning increment. The values of FWHM decreased with increasing *T*_*sub*_ for *T*_*sub*_ < 150 °C and become saturated for *T*_*sub*_ ≥ 150 °C. The smallest FWHM of 0.027 ± 0.008° (and hence the highest crystallinity of the constituent grains) was obtained for the film deposited at *T*_*sub*_ = 150 °C. This observation was consistent with the top-view SEM pictures as shown in Fig. 3, where clear facets of crystalline grains emerged in the coumarin-6 films deposited at *T*_*sub*_ ≥ 150 °C.

Because the intensity of (010) peak was too strong (especially for those coumarin-6 films deposited at *T*_*sub*_ ≥ 150 °C), in linear scale most of the other peaks were suppressed. To reduce the strong (010)-orientation effect from the as-deposited films, the powder-samples were taken from the corresponding films and their XRD results are presented in Fig. 6(b) with logarithmic scale. For a higher *T*_*sub*_ (150 or 165 °C, as comparing with 125 °C), the diffraction intensities of the main peaks and of the higher indexed planes were more clearly detected due to the larger surface diffusion energy of the adhered molecules in the growing interface. This finding is consistent with the facts that clear facets were observed for *T*_*sub*_ ≥ 150 °C as shown in Fig. 3. By performing the general structure analysis system (GSAS)^27^ on these three spectra in Fig. 6(b), the triclinic unit cell volume was estimated to be 863.8 ± 2.1 Å^3^ for *T*_*sub*_ = 125 °C, 858.8 ± 0.2 Å^3^ for *T*_*sub*_ = 150 °C, and 857.3 ± 0.3 Å^3^ for *T*_*sub*_ = 165 °C, separately. The packing density of the coumarin-6 molecular crystalline grains became slightly compact for *T*_*sub*_ ≥ 150 °C. The lattice parameters averaged from the grains with high crystallinity fabricated at *T*_*sub*_ = 150 and 165 °C were obtained as followings: *a* = 8.965 ± 0.005 Å, *b* = 11.139 ± 0.002 Å, *c* = 8.920 ± 0.007 Å, *α* = 95.13 ± 0.02°, *β* = 104.56 ± 0.01°, and *γ* = 86.72 ± 0.08°. These data are in agreement with small crystals growth from supersaturated solutions^1,14^.

**Figure 7:**
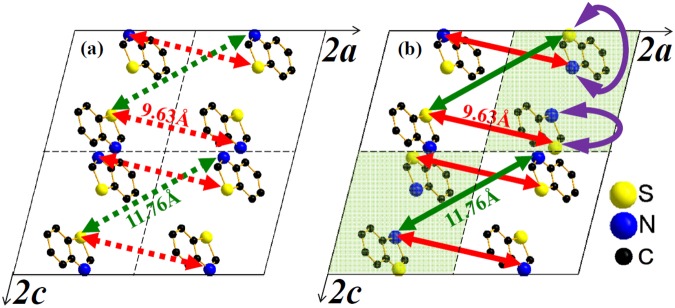
Simulated unit cells built in a double-lattice-constant basis from GSAS. For clarity, only the benzothiazolyl moiety and projection on the *ac* plane are shown. (**a**) The projected 2 × 2 unit cells are identical without isomer-effect consideration. (**b**) By exchanging the S and N atoms in the benzothiazolyl moiety of the unit cells (indicated with a background of light-green color) to symbolize the change between S- and N-isomers, then the atoms at both ends of the green and red arrows become identical to lead constructive interference peaks as observed in Fig. 6(b).

Interestingly, two more peaks at 2*θ*~7.410° and 9.022° (with a fitted FWHM = 0.126° and 0.075°, correspondingly) in Fig. 6 remain for further discussion. The latter was observable even in linear scale for *T*_*sub*_ < 142 °C. By using GSAS with zero-check correction, the spacings of parallel planes corresponding to these two peaks were calculated to be *d*_1_ = 11.89 ± 0.10 Å (which exceeds the longest *b*-axis length) and *d*_2_ = 9.77 ± 0.04 Å, respectively. To the best of our knowledge, these two peaks have not been reported in the literatures. As mentioned earlier (see Fig. 1), within the coumarin-6 crystalline grains the S- and N-isomers should be distinguished. Due to the random coexistence of the S- and N-isomers within the grains, a reasonable lattice periodicity should be twice or more than the original one. Figure 7 displays the simulated unit cells built in a double-lattice-constant basis from GSAS with isomer-effect consideration. For clarity, only the benzothiazolyl moiety and projection on the *ac* plane are shown. As illustrated in Fig. 7, the green and red lines characterize two kinds of simulated distances of atom pairs, 11.76 Å and 9.63 Å, which closely matched to the experimentally XRD derived-spacings of *d*_1_ and *d*_2_ in Fig. 6(b), respectively. Figure 7(a) depicts the crystal structure without isomer-effect consideration, *i.e*., the projected 2 × 2 unit cells are identical. The constructive interference cannot be effectively formed by heterogeneous (S and N) atoms in each pair (connected by dotted arrows). However, by exchanging the S and N atoms of the benzothiazolyl moiety in the cells indicated with a light-green background color in Fig. 7(b) to symbolize the exchange between S- and N-isomers, the atoms at both ends of the green and red solid lines become identical. Thus, the constructive interference can be formed by the homogeneous (S or N) atoms in each pair. One final message from Fig. 6(b) is that for *T*_*sub*_ ≥ 150 °C a weak tail with exponential decay with increasing 2*θ* after the (010) peak was observed. This phenomenon can be explained in terms of the small interlayer-spacing fluctuations which is commonly detected in two dimensional systems^28,29^. Thus, the strongly preferred growth direction along (010) plane (which is nearly parallel to the long axis of coumarin-6 molecule as depicted in Fig. 6) might induce a two dimensional characteristics for the coumarin-6 polycrystalline films deposited with *T*_*sub*_ ≥ 150 °C.

### Optical properties of coumarin-6 polycrystalline films

**Figure 8:**
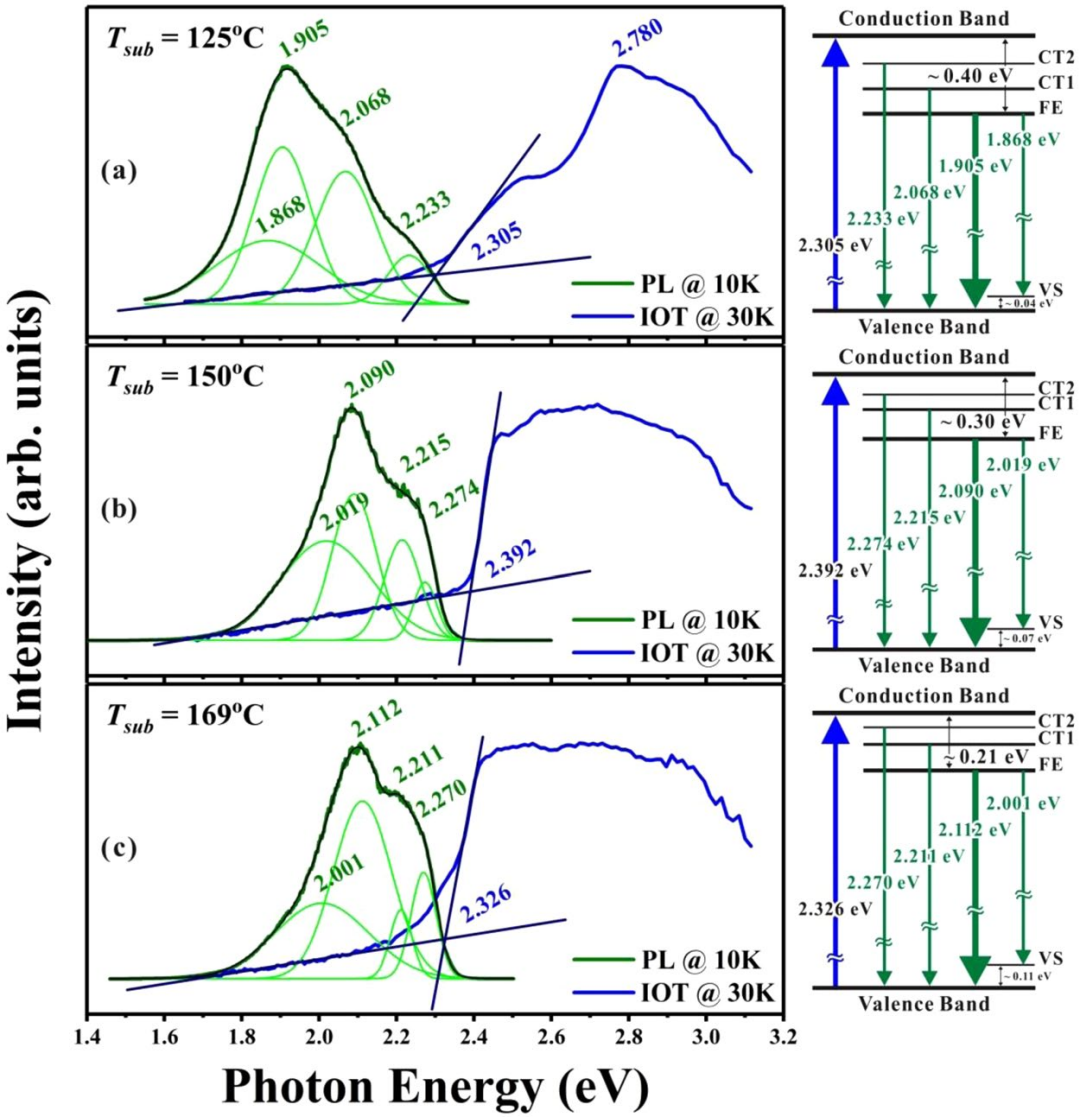
Comparison of the normalized PL and IOT spectra performed at low *T* for the coumarin-6 polycrystalline films deposited at *T*_*sub*_ = (**a**) 125, (**b**) 150 and (**c**) 169 °C with *T*_*sou*_ = 185 °C. The schematic energy-term diagrams on the right-side summarize the corresponding transitions for *E*_*op*_ and for the four PL peaks.

As vaporized small organic molecules condense into an organic molecular solid film, the close pack of splitting electronic levels due to intermolecular interactions forms a continuous electronic band^17^. However, in contrast to covalent-bonded inorganic solids, the relatively weak intermolecular interactions in organic molecular solids usually result in a small bandwidth of around 0.5~1.0 eV. Furthermore, the magnitude of optical bandgap (*E*_*op*_) in an organic crystalline solid is expected to be smaller than the energy difference between the lowest unoccupied molecular orbital (LUMO) and the highest occupied molecular orbital (HOMO) of its isolated molecule^17^. From the inverse of optical transmission (IOT) spectrum (which is associated with the absorption spectrum), *E*_*op*_ can be experimentally deduced. Figure 8 depicts three IOT and PL spectra for coumarin-6 polycrystalline films fabricated with *T*_*sub*_ = 125, 150, and 169 °C. The absorption threshold (*i.e*., *E*_*op*_) was practically defined at the intersection of two guide lines^25^, one for the transmitted background with *hv* ≪ *E*_*op*_ and the other one for the strong absorption with *hv* > *E*_*op*_. For the film deposited at *T*_*sub*_ = 150 °C, the abrupt increase in IOT spectrum (with smallest band-tail absorption) around *E*_*op*_ suggests that this film had a highest crystallinity among other films, and this deduction was consistent with its smallest FWHM detected from Fig. 6(a). Because of its asymmetric molecular structure, coumarin-6 molecules possess a permanent dipole^14^, and the strong dipole-dipole interactions may enhance the packing of the adhered molecules. The best crystallinity of coumarin-6 grains observed for *T*_*sub*_ = 150 °C could be due to a compromise between the increase of the surface diffusion energy for *T*_*sub*_ < 150 °C and the thermal agitation of the adhered molecules for *T*_*sub*_ > 150 °C. Thus, the highest *E*_*op*_ = 2.392 ± 0.007 eV (with a least band-tail effect) was determined from the coumarin-6 films deposited at *T*_*sub*_ = 150 °C. Moreover, only for the thin enough films deposited at *T*_*sub*_ ≤ 125 °C, the molecular characters can be revealed in the IOT spectra, and a weak absorption peak around 2.768 ± 0.008 eV was assigned to the HOMO-LUMO transition, which is also consistent with the published results obtained for coumarin-6 molecules dissolved in various solvents^5^.

Complementary to IOT spectra, Fig. 8 compares three normalized PL spectra for coumarin-6 polycrystalline films fabricated at *T*_*sub*_ = 125, 150, and 169 °C. All the PL spectra can be decomposed into four components by a Gaussian fit with the main peak being assigned to the Frenkel exciton (FE) emission. Accordingly, the weak peak with energy lower than that of FE was attributed to the participation of its molecular vibrational states (VS) because of the rich vibrational motion in coumarin-6 molecule. The other two with energies higher than that of FE were assigned to the charge transfer excitons (CT1 and CT2) in which the electron-hole distances were assigned to the distances between the nearest and next-nearest neighbors of its constituent molecules, respectively. The emergence of CT1 and CT2 might be related to the coexistence of S- and N-isomers within courmarin-6 crystalline grains, which remains for further study. The schematic energy-term diagrams as shown in the right-side of Fig. 8 illustrate the corresponding transitions for the threshold of absorption (*E*_*op*_) and for the four PL peaks (with the main peak assigned to FE emission). For FE in the molecular solid films, the electron-hole pair is localized on the same molecule^17^. Considering the intermolecular interactions, the energy for FE emission and *E*_*op*_ in molecular solid films should be different from that in an isolated molecule and are expected to be affected by the packing of its constituent molecules. Hence, as depicted in Fig. 8, *E*_*op*_ and the energy positions of PL peaks for coumarin-6 polycrystalline films were varied with *T*_*sub*_, which is consistent with the fact that the unit cell volume simulated by GSAS from the data of Fig. 6(b) were slightly altered with respect to *T*_*sub*_ as mentioned above.

## Conclusion

Vacuum deposition technique was adopted to deposit coumarin-6 polycrystalline films with *T*_*sou*_ determined by a conventional TGA and *T*_*sub*_ chosen from a CSS method. Various coumarin-6 films were fabricated with *T*_*sub*_ from 106 to 178 °C with a fixed *T*_*sou*_ at 185 °C. Because of its slenderer and more asymmetric structure accompanied by a larger permanent dipole, the adhered coumarin-6 molecule on top of the growing interface encounters a higher steric hindrance to occupy the appropriate location and correct orientation, and hence possesses a larger steric energetic barrier of 0.92 eV as estimated from the Arrhenius plot of growth rate versus 1/*T*_*sub*_. From top-view SEM pictures, for *T*_*sub*_ < 150 °C, the as-deposited coumarin-6 thin films exhibit a twisted pattern and a kinematic roughness; while for *T*_*sub*_ ≥ 150 °C, clear facets emerge due to the increase of surface diffusion energy. From XRD spectra, a protruding peak assigned to (010) peak reveals that the preferred growth direction of these films on ITO substrate is nearly parallel to the long axis of coumarin-6 molecule. From an analysis of FWHM of the (010) peak, the polycrystalline film deposited at *T*_*sub*_ = 150° exhibits a highest crystallinity among others due to a compromise between surface diffusion and thermal agitation of the adhered molecules. In addition, to account for the two noticeable peaks at 2*θ*~9.007° and 7.260°, the lattice constants of the triclinic cell should be doubled due to the co-existence of two coumarin-6 isomers, namely, the N-isomer and S-isomer, which are introduced in coumarin-6 crystalline grains for the first time. Furthermore, the bandgap of the coumarin-6 film deposited at *T*_*sub*_ = 150 °C from OT is estimated to be around 2.392 eV. From PL spectra, four components are obtained by a Gaussian fit. The main peak is assigned to the FE emission; a weak peak with energy lower than that of FE is recognized to the participation of the molecular vibrational states; and the other two with energies higher than that of FE are attributed to charge transfer excitons.

## Methods

### Fabricating of coumarin polycrystalline films

The vacuum deposition system together with the growth ampoule adopted by this work were described in details in our previous papers^21–25^. As demonstrated in Fig. 1, an ITO coated glass was chosen as the substrate and adhered underneath a graphite susceptor. A thermocouple was inserted from the other side of the graphite susceptor to monitor *T*_*sub*_ *in situ* during the whole process. The temperature profile adopted by *T*_*sou*_ was pre-measured by inserting a thermocouple at the bottom of the growth ampoule where the source powder should be located. Due to the different ways of measurement, the steady state temperature profiles adopted for *T*_*sou*_ and *T*_*sub*_ were slightly shifted as depicted in Fig. 1. Coumarin-6 source powder (with purity > 98.0% from Tokyo Chemical Industry Co., Japan) was used as-received and placed at the bottom of the ampoule. By adjusting the positions of the substrate site relative to the bottom of the ampoule with respect to the temperature profile in a vertical furnace, *T*_*sou*_ and *T*_*sub*_ could be controlled separately as shown in Fig. 1. After a typical cleaning process, the whole growth ampoule was assembled and loaded with 40–50 mg coumarin-6 source powder, and then kept at room temperature under dynamic vacuum of 5 × 10^−2^ Torr for 1.0 h to remove residue moisture and vaporized impurities. Next, the growth ampoule was put into a vertical furnace for a preheat process to warm-up the substrate, in which the substrate site was located on a hotter spot with source powder on a colder side for 6~15 min to allow *T*_*sub*_ approaching the selected value. After the preheat treatment, the ampoule was pull up to the designated position for vacuum deposition, and it took another 9 ± 1 min for the source powder to reach 95% of the chosen *T*_*sou*_ value (in Kelvin temperature scale). The deposition period *τ* was maintained for 51 ± 1 min under dynamic vacuum condition. At the end of deposition, the growth ampoule was furnace-cooled to room temperature without dynamic vacuum.

### Characterization

TGA experiment of coumarin-6 powder was carried out by using a TA Instrument TGA Q50 analyzer under a flow of nitrogen gas with a heating rate of 10 °C/min. The surface morphology as well as the thickness of the as-deposited coumarin-6 polycrystalline film was taken by a JOEL JSM-6335F field emission scanning electron microscopy (SEM). X-ray diffraction (XRD) analysis on coumarin-6 polycrystalline film or powder was performed on a Bruker D8-Advance with Cu-*K*α emission and with a scanning increment of 0.0149°. Optical transmission (OT) through coumarin-6 film was taken by a scanning monochromator (ARC SpecrroPro-500) with a tungsten-halogen lamp as the light source. The optical transmittance intensity *I*_*OT*_ and the original incident light intensity *I*_0_ were measured by an optometer (Graseby UDT S370) with wavelength correction. Then, the normalized OT spectrum was obtained by dividing *I*_*OT*_ by *I*_0_ at each photon energy position. PL spectrum on the coumarin-6 film was performed with exciting by a focused diode laser of 403 nm. The collected luminescence from coumarin-6 film was dispersed by a spectrometer (Jobin Yvon/Spex TRIAX 550) and then detected with a photomultiplier tube. The temperature of the sample site during the OT and PL measurements was controlled by a cryostat system.

## References

[1] Jasinski JP, Paight ES (1995). 3-(2-Benzothiazolyl)-7-(diethylamino)-coumarin. Acta Cryst. C.

[2] Edetsberger M (2011). Effective staining of tumor cells by coumarin-6 depends on the stoichiometry of cyclodextrin complex formation. J. Incl. Phenom. Macrocycl. Chem..

[3] Lakner PH (2017). Applying phasor approach analysis of multiphoton FLIM measurements to probe the metabolic activity of three-dimensional *in vitro* cell culture models. Sci. Rep..

[4] Gu G, Ong PP, Li Q (1999). Photoluminescence of coumarin 540 dye confined in mesoporous silica. J. Phys. D: Appl. Phys..

[5] Raikar US (2006). Solvent effects on the absorption and fluorescence spectra of coumarins 6 and 7 molecules: Determination of ground and excited state dipole moment. Spectrochim. Acta A.

[6] Nedumpara RJ (2007). Study of solvent effect in laser emission from coumarin 540 dye solution. Appl. Optics.

[7] Tang CW, VanSlyke SA, Chen CH (1989). Electroluminescence of doped organic thin films. J. Appl. Phys..

[8] Hebner TR, Wu CC, Marcy D, Lu MH, Sturm JC (1998). Ink-jet printing of doped polymers for organic light emitting devices. Appl. Phys. Lett..

[9] Jiang X (2000). Doped organic light-emitting diodes based on random copolymers containing both hole and electron transport groups. Mater. Res. Soc. Symp. Proc..

[10] Huang Y-S, Jou J-H, Weng W-K, Liu J-M (2002). High-efficiency white organic light-emitting devices with dual doped structure. Appl. Phys. Lett..

[11] Cheng J-A, Chang C-P, Chen C-H, Lin M-S (2005). The fluorescent quantum efficiency of copolymers containing coumarin-6 at the side-chain. J. Polym. Res..

[12] Uddin A, Lee CB, Wong J (2011). Emission properties of dopants rubrene and coumarin 6 in Alq_3_ films. J. Lumin..

[13] Lou Y, Geng Z, Wang Z, Naka S, Okada H (2012). Improvement of roll-off in power efficiency for mixed single layer organic light emitting devices by co-doping. Synth. Metals.

[14] Li X, Son Y-A (2010). C–H···π and C–H···O Interactions in coumarin 6: 3-(2-benzothiazolyl)-7-(diethylamino)-coumarin. Text. Coloration Finish..

[15] Laudise RA, Kloc C, Simpkins PG, Sigriest T (1998). Physical vapor growth of organic semiconductors. J. Cryst. Growth.

[16] Forrest SR (2004). The path to ubiquitous and low-cost organic electronic appliances on plastic. Nature.

[17] Schwoerer, M. & Wolf, H. C. *Organic Molecular Solids* (Wiley-VCH, Weinheim, Germany, 2005).

[18] Price DM, Hawkins M (1998). Calorimetry of two disperse dyes using thermogravimetry. Thermochim. Acta.

[19] Price DM (2001). Vapor pressure determination by thermogravimetry. Thermochim. Acta.

[20] Chen R-S, Lin Y-J, Su Y-C, Chiu K-C (2001). Surface morphology of C_60_ polycrystalline films from physical vapor deposition. Thin Solid Films.

[21] Cheng W-R, Tang S-J, Su Y-C, Lin Y-J, Chiu K-C (2003). Effects of substrate temperature on the growth of C_60_ polycrystalline films by physical vapor deposition. J. Cryst. Growth.

[22] Jan D-J (2011). Growth and characterization of tris(8-hydroxyquinoline)-aluminum molecular films. Thin Solid Films.

[23] Chiu Y-C, Chen B-H, Jan D-J, Tang S-J, Chiu K-C (2011). Growth behavior of CuPc films by physical vapor deposition. Cryst. Res. Technol..

[24] Lin K-Y (2016). Rubrene polycrystalline films growth from vacuum deposition at various substrate temperatures. J. Cryst. Growth.

[25] Lin K-Y (2017). Role of molecular conformations in rubrene polycrystalline films growth from vacuum deposition at various substrate temperatures. Sci. Rep..

[26] Markov, I. V. *Crystal Growth for Beginners* (World Scientific, Singapore, 1995).

[27] Larson, A. C. & von Dreele, R. B. *GSAS General Structure Analysis System* (Los Alamos National Laboratory, New Mexico, USA, 1994).

[28] Shi H, Reimers JN, Dahn JR (1993). Structure-refinement program for disordered carbons. J. Appl. Cryst..

[29] Zhou Z, Bouwman WG, Schut H, Pappas C (2014). Interpretation of X-ray diffraction patterns of (nuclear) graphite. Carbon.

